# A Systematic Review of Qualitative Studies Investigating Motives and Experiences of Recipients of Anonymous Gamete Donation

**DOI:** 10.3389/fsoc.2022.746847

**Published:** 2022-02-16

**Authors:** Tobias Bauer

**Affiliations:** Faculty of Humanities and Social Sciences, Kumamoto University, Kumamoto, Japan

**Keywords:** gamete donation, sperm donation, oocyte donation, donor anoymity, sociology of ignorance, systematic review

## Abstract

The decision to use an anonymous gamete donation in fertility treatment could have significant long-term psychological and social effects for all stakeholders involved. In light of the growing recognition of donor-conceived children’s right to know their genetic parentage, this entails profound ethical implications. This review aims to carve out the full spectrum of recipients’ motives and experiences related to donor anonymity which could serve as an analytical framework for future ethical and sociological research on issues of donor anonymity. This review was conducted following a seven-step approach for systematic reviews of empirical bioethics literature. The characteristics and quality of the studies included in this review were reported. Data analysis was conducted using qualitative content analysis and was informed by sociological functionalist theorizations of ignorance. The 53 studies selected showed a diverse spectrum of characteristics concerning date and country of study, methodology, family type of participants, sample size, and the timing of data collection in relation to the stage of treatment. A total of 22 categories of motives and experiences of recipients concerning donor anonymity were identified inductively and grouped into five main categories. Donor anonymity was identified as a eufunctional form of ignorance, by which the recipients experienced or intended to control, regulate, or protect inter-stakeholder relations. Interpreting recipients’ motives and experiences concerning donor anonymity as a form of ignorance directed toward particular stakeholders helps reframe the discourse on donor anonymity. It is a fruitful approach that can be refined further and applied in future research. This review identified possible directions for future investigations on motives for donor anonymity: the need for more thorough inquiries into the change in recipients’ preferences over time, such as in the form of longitudinal studies and research on the perspective of non-biological parents.

## 1 Introduction

Choosing to conceive a child through a sperm or oocyte donation is a significant decision for aspiring parents to make in their quest for parenthood. However, such a decision would also lead them to make important choices around areas such as the donor type, that is, whether to use a known, anonymous, or identity-release donor. A known donor, as the name suggests, is known to the recipient at the time of treatment, such as in the case of oocyte donation by a sister of the recipient. An anonymous donor is one whose identity is not known to the recipient—such as in the case of a sperm donation via a sperm bank in a country with mandatory donor anonymity, where only non-identifiable information is passed on to the recipients. An identity-release donor is one whose identifying information will be released to the (adult) child upon request. The implications of the choice of donor type such as possible ethical dilemmas and potential long-term psychological and social effects for the recipients themselves, the donor-conceived offspring, the donor, and the relationship among these stakeholders are subject of an ongoing debate (McWhinnie, 2001; de Melo-Martín, 2014; Freeman et al., 2014; Golombok et al., 2017; Pennings, 2021).

Along with a growing recognition of the rights of donor-conceived children to know their genetic parentage, donor anonymity has been coming under increasing scrutiny in a growing number of jurisdictions since the 1980s (Allan, 2018). As a result, the recipients’ choice of opting for anonymous gamete donation in the course of treatment by means of assisted reproductive technology (ART) may be restricted by the policies and regulations of medical institutions providing the treatment, or by legal constraints such as mandatory donor registries in some countries (e.g., in the case of sperm donation in Sweden, the United Kingdom, New Zealand, and Germany). In contrast, in countries like Spain and the Czech Republic, anonymous donations are mandatory and protected by law.

However, in many cases, prospective recipients of gamete donations are *de facto* in a position to choose between an anonymous and non-anonymous donation. This may be because the recipients reside in a jurisdiction that imposes no legislative constraints on their choice concerning donor anonymity (such as in the US). For example, it has been suggested that many recipients of oocyte donation in the US “have strong preferences regarding the use of anonymous versus directed donors and will express and exercise these preferences when given the opportunity” (Greenfeld et al., 1998:1,013). Some recipients resort to the emerging sector of cross-border reproductive services (CBRS) as a means of broadening the range of fertility options available to them, including the choice between anonymous and non-anonymous donations for their ART treatment (Bergmann, 2011). The desire to use an anonymous donor is reported as one of the possible motivations influencing the decision to consider the use of CBRS (Rodino et al., 2014; Hertz et al., 2016; Simopoulou et al., 2019). A rising online market facilitating contact between potential sperm donors and recipients in an informal setting beyond institutional and state regulations provides potential self-insemination recipients with opportunities to freely exercise and negotiate their preferences concerning donor (non-)anonymity, the extent of information on the donor, and the level of involvement with the donor, thus circumventing legal restrictions and blurring “the distinction between categories of ‘anonymous’, ‘known’ and ‘identity release’ donations” (Jadva et al., 2018:112).

Studies quantitatively reporting recipients’ preferences and actual choices concerning donor (non-)anonymity cover a broad spectrum of countries, timeframes, and samples in various settings and accordingly present strongly varying findings (Stuart-Smith et al., 2012). The first report of the National Lesbian Family Study focusing on the US, for example, found that 47% of the participating lesbian mothers preferred anonymity of their sperm donor (Gartrell et al., 1996), whereas a study at a Dutch fertility center offering both anonymous and identifiable donations reported that 37% of heterosexual couples and only 2% of lesbian couples chose anonymous sperm donors (Brewaeys et al., 2005). A study on heterosexual oocyte recipients at a Belgian clinic found that 69% of the participants opted for forms of anonymous donation (Laruelle et al., 2011), and a global survey of 1700 recipients of sperm donations conducted by the Donor Sibling Registry reported that 73% of the respondents had used an anonymous donor (Sawyer et al., 2013).

A thorough understanding of motives and preferences, and the experiences and retrospective reflections related to the recipients’ choice between a known, anonymous, and identity-release donor plays a crucial role in the following four contexts.

First, since the 1980s many countries have observed a growing trend toward a policy of increased openness in ART involving gamete donation, both in the form of encouraging disclosure of the mode of conception to the donor-conceived offspring as well as promoting legislative measures to ban donor anonymity (Allan, 2018). This legislative trend started with Sweden, which in 1985 became the first country to remove donor anonymity, and is still ongoing with Germany implementing its Sperm Donor Registry Act as recently as 2018. The major driving force behind this development is considered to be the increasing acknowledgment of the right of donor-conceived children to know their genetic parentage, which involves being informed about one’s status of being donor-conceived and being granted access to both non-identifying and identifying information about the donor. This right is in turn underpinned by a growing recognition of the impact that the denial of this information could have on the process of identity formation of a donor-conceived child and the importance to have access to one’s genetic parents’ medical history (Allan, 2018). In many jurisdictions, the process of implementing policies and regulations banning donor anonymity is triggered or reinforced by the voices of individual or organized donor-conceived persons demanding a recognition of their rights. In the political and legal discourses on donor anonymity, the issue of donor anonymity tends to be framed as a conflict between the recipients’ rights to privacy and autonomy on the one hand and their children’s right to know their genetic origins on the other (Frith, 2001; Herbrand and Hudson, 2015). Accordingly, recipients’ choices for anonymous donation tend to be perceived as being diametrically opposed to the well-being of the child as expressed in the assessment that “in the current discourse, it is frequently suggested that the practice of anonymization and secrecy mainly serves the ‘egoistic’ interests of the paternal couple and the commercial goals of fertility doctors—at the expense of the affected children” (Wehling, 2015:109). Here, a thorough inquiry into the whole spectrum of reasons recipients might have to prefer donor anonymity could benefit the debate in suggesting possible approaches to reconcile the interests of recipients and donor-conceived children.

Second, in many such cases, public perception and the political deliberation process tend to be determined by a focus on the perspective of the donor-conceived offspring and on donor-related issues, such as the question of how to prevent a decline in gamete donation, which was assumed would occur following the abolition of donor anonymity. The perspective of recipients, however, seems far less represented in these discourses (Turkmendag et al., 2008) and preceding research has pointed out stagnating rates of disclosure of the mode of conception by the parents to their offspring, despite bans on donor anonymity. For example, in Sweden, the first country to remove donor anonymity, a discrepancy between high rates of recipients’ intentions to disclose and a significantly lower rate of actual disclosure was reported (Lalos et al., 2007; Isaksson et al., 2012). Here, a deeper understanding of the various reasons why some aspiring parents prefer anonymous gamete donations would be highly instructive for policymakers involved in passing legislation concerning issues of donor anonymity. It would help them understand the needs and concerns of various groups of gamete recipients to find informed and well-balanced solutions considering both the rights and needs of all stakeholders involved. As research on the influence of the use of identifiable and anonymous gamete donation on recipients’ disclosure patterns has been inconclusive thus far (Laruelle et al., 2011; Freeman et al., 2012; Stephenson et al., 2012; Greenfeld and Klock, 2004), a more thorough and in-depth understanding of the aspiring parents’ preferences for identifiable or anonymous gamete donation and their underlying motives can contribute toward clarifying the relationship between deciding the type of donor and attitudes toward the disclosure of donor identity.

Third, pre-treatment psychological counseling is an important part of many ART programs based on gamete donation. Effective counseling with the aim of sensitizing aspiring parents to the implications of their choices concerning donor anonymity is closely related to an understanding of the concerns of aspiring parents in relation to donor identifiability and anonymity as well as intra-familial mechanisms and dynamics potentially triggered by the recipients’ choice of donor type. A comprehensive understanding of the full range of patterns, motives, and mechanisms underlying the recipients’ preferences concerning donor anonymity can be considered instructive in offering the recipients successful psychological guidance throughout the challenging decision-making process.

Fourth, the phenomenon of CBRS puts aspiring parents in a position to have extended options concerning their choice from among the options available to them. Although there are various factors underlying a decision to seek treatment abroad, such as the type of treatment sought, the type of recipient, the country of origin and destination and their disparities concerning the availability of donors, waiting times, treatment costs, or success rates (Hudson et al., 2011), some findings in the literature also suggest that some “fertility tourists” opt for treatment abroad explicitly because of the availability of anonymous donations (Bergmann, 2011; Hertz et al., 2016). It can therefore be presumed that the motives and experiences of recipients concerning donor anonymity are also crucial for an accurate understanding and analysis of the phenomenon of CBRS.

Research has shed light on a number of important aspects of recipients’ attitudes and preferences concerning donor anonymity in country-specific contexts by focusing on various recipients such as heterosexual or lesbian couples or single mothers by choice. A systematic overview of the possible motives and experiences of recipients from a more universal perspective remains, to the best of the author’s knowledge, a desideratum. Therefore, this review aims to carve out the full spectrum of recipients’ motives and experiences related to donor anonymity by a systematic examination of the existing qualitative research. Intended to be a preliminary step to further investigation, this review does so by proposing a theory-driven approach that could serve as an analytical framework in future ethical and sociological research on issues of donor anonymity.

## 2 Methods

This systematic review was conducted following the seven-step approach proposed by Strech et al. (2008a). This approach has proven successful in assessing research on stakeholders’ attitudes in other contexts in the thematic field of bioethics, such as physician attitudes toward advanced directives (Coleman, 2013), attitudes of medical personnel and the public toward organ donation after cardiac death (Bastami et al., 2013), and attitudes of patients, families, and healthcare providers toward medical futility (Müller and Kaiser, 2018). As a systematic review of empirical bioethics literature (McDougall, 2014), this review collates qualitative studies inquiring into the motivations and experiences of gamete recipients related to their concrete choices of an anonymous donor, while taking into account that these choices are framed by specific legal, institutional and medical settings as a result of bioethical deliberation, and by a multitude of distinct societal and cultural factors.

### 2.1 Review Question

The review question was specified using the methodology, issues, participants (MIP) model outlined by Strech et al. (2008a). Studies with a significant focus on qualitative inquiry (methodology), exploring recipients’ (participants) attitudes toward, motives for, and experiences with choosing anonymous sperm or oocyte donation (issues) were included. The review question was: “What motives and experiences of recipients choosing anonymous gamete donation are reported in the relevant qualitative research?”

### 2.2 Selection of Databases

Based on the results of preliminary scoping searches and on the understanding that research relevant to the review question would be situated at the intersections of multiple disciplinary fields like psychology, sociology, and medical ethics, I decided to pursue a broad approach to identify studies from a wide range of backgrounds. Therefore, the following seven databases covering a broad spectrum of disciplines were included in the literature search: Web of Science Core Collection, SCOPUS, MedLine, PubMed, CINAHL, PsycINFO, and ProQuest Central.

### 2.3 Development of the Search Algorithm

After the initial preliminary scoping searches conducted on Web of Science and MedLine, the review question was translated into a search syntax comprising four clusters relating to the topics of 1) anonymity, 2) gamete, 3) donation, and 4) motives. The 2–15 keywords in each cluster were connected using the Boolean operator OR, while the four clusters were connected using the Boolean operator AND. The search syntax was developed and refined through an iterative process using five key references identified in the preliminary scoping searches to check search syntaxes on sensitivity and specificity (Baetens et al., 2000; Brewaeys et al., 2005; Frith et al., 2012; Stuart-Smith et al., 2012; de Melo-Martín et al., 2018). Owing to the multidisciplinary character of the topic and based on the results of the preliminary scoping searches, it was decided that MeSH terms would not be used, and a high sensitivity search syntax would be prioritized instead. The search syntax was adjusted according to the interface and search options available in each of the databases used (Supplementary File S1). The studies identified were merged and stored in the reference management software CITAVI.

### 2.4 Ancillary Search Strategies

The search of bibliographic databases was supplemented by a manual search of major journals, and forward and backward citation chaining of key references. However, this process did not reveal any additional studies that had not been identified using the initial search algorithm.

### 2.5 Relevance Assessment

The references retrieved were assessed for inclusion or exclusion. Empirical studies providing original qualitative data by means of interviews, fieldwork, questionnaires or online surveys including open-ended questions, and mixed-method studies with a significant focus on qualitative inquiry were included. The studies included involved (potential or actual) recipients of sperm or oocyte donation, and were focused on their (and their partners’) attitudes, experiences, preferences, and/or motivations for considering the usage of anonymous gamete donations. Studies either focused on the pre-treatment decisions of potential recipients or retrospective accounts of recipients reflecting on their former choices concerning donor anonymity and the experiences that resulted therefrom. Alternatives to choosing anonymous donors may have been the use of known donors (e.g., friends or relatives) and non-related non-anonymous donors (e.g., provided by a sperm/oocyte bank), or donations involving non-absolute forms of anonymity, such as identity-release donations. Special forms of known-anonymous oocyte donation where recipients provide a donor recruited among fertile friends or family members to the program and whose oocytes are shared anonymously with other participants (Frydman et al., 1990) were considered anonymous for the purpose of this study. Anonymous sperm donation was not defined as being limited to treatment in medicalized settings, but was considered to also include anonymous donations procured for private self-insemination. Studies on participants who *de facto* did not have an actual choice concerning donor anonymity because of legal, regulatory, financial, or other restrictions, but nevertheless inquired into the recipients’ (hypothetical) considerations concerning anonymous gamete donation were also included. Studies on recipients of non-anonymous donations were also included if they discussed the recipients’ decision-making process around donor anonymity. Purely quantitative, non-empirical, and studies published in languages other than English, German, or French were excluded. Studies focusing solely on embryo or mitochondrial donation were also excluded. This review was limited to (aspiring) parents (and their partners) who were at least already in the process of starting treatment. Thus, studies drawing on surveys of sections of the general population were excluded.

### 2.6 Quality Assessment

The quality assessment of qualitative research is subject to an ongoing debate. Recent research suggests that there is still no consensus on the necessity, value, methods, and criteria of quality assessment of qualitative research in the context of systematic reviews. The “[e]xclusion of so-called inadequately reported studies had no meaningful effect on the synthesis” (Carroll et al., 2012:1,425). Therefore, in accordance with previous syntheses of qualitative research (Thomas and Harden, 2008; Atkins et al., 2008), I decided to conduct quality assessment of the literature that had passed the relevance assessment process in order to report on the quality and characteristics of the studies included in this review. However, this was done without excluding any studies for reasons of poor quality. Drawing on the experiences of previous systematic reviews in empirical bioethics (Strech et al., 2008a), an adapted version of the CASP criteria developed for the critical appraisal of qualitative research was selected for quality assessment (Strech et al., 2008b; Critical Appraisal Skills Programme, 2018).

### 2.7 Data Analysis

In order to answer the review question at a high level of abstraction, data analysis was informed by sociological accounts of the functions of ignorance and the intentionality of knowledge restriction. Assuming that donor anonymity constitutes a form of non-knowledge or ignorance (Funcke, 2013; Wehling, 2015), recipients’ expectations and experiences in relation to donor anonymity can be linked to sociological functionalist theorizations of ignorance, in which ignorance “must be viewed not simply as a passive or dysfunctional condition, but as an active and often positive element in operating structures and relations” (Moore and Tumin, 1949:795). Data analysis was therefore conducted on the premise that donor anonymity can be interpreted as a “eufunctional” form of ignorance (Schneider, 1962), which means that this specific form of non-knowledge can be analyzed as potentially being beneficent for the well-being of the stakeholders, the stability of the family and as having the potential to positively affect the relations between the stakeholders involved in gamete donation settings, circumventing the negative effects alternative donor types such as known or identifiable donors might have on family-making. This concept of eufunctional ignorance on the analytical level corresponds to the recipients’ experience or intention to control, regulate, or protect inter-stakeholder relations by their choice of an anonymous donation.

Among the various methods of analyzing and synthesizing qualitative data in the context of systematic reviews (Dixon-Woods et al., 2005), applying qualitative content analysis based on the procedures proposed by Mayring (2015) was identified as a promising approach for this review. This is a flexible tool adaptable to the specific requirements of qualitative analysis of stakeholder attitudes in the context of a systematic literature review. It further allows for a mixed approach (deductive-inductive) and a theory-guided synthesis in literature reviews.

Adapting Mayring’s procedural model, which proposes a segmentation of the analytical process into six consecutive steps (Mayring, 2015:85–87), inductive development of categories was orientated toward the review question. I started out by 1) defining the authors’ analytical reproductions of the participants’ accounts (including the cited interview data) of concrete motives, preferences, and experiences with regard to anonymous gamete donation as the material to be screened in this review. 2) Sub-categories were defined as describing the concrete means facilitated by donor anonymity by which the recipients controlled, regulated, or protected inter-stakeholder relations (i.e., the anticipated or experienced eufunction of ignorance in the form of donor anonymity). These were then aggregated into categories on an enhanced level of abstraction where possible. 3) After developing and constantly refining the inductive codes, sub-categories, and categories of 25 of the retrieved studies, using NVivo software, coding rules, sub-categories, and categories were 4) reviewed and 5) applied to the entire material. 6) To interpret the results, I followed Mayring’s approach of deductive introduction of theory by grouping the categories that had emerged from the data into five overarching main categories derived from theoretical considerations related to the sociology of ignorance. Following recent sociological examinations of the “intentionality” of ignorance, stating that ignorance can be the result of a conscious, deliberate choice to keep oneself (do-not-want-to-know) or others (do-not-want-to-let-know) in a state of non-knowing (Wehling, 2006; Hertwig and Engel, 2020), these main categories were based on which of the stakeholders the recipients intended to keep ignorant of the donor identity (or the child’s identity) by choosing an anonymous donor: the recipients themselves, the child, the donor, and or the family environment.

Before data analysis, the studies were screened for their major characteristics. An adequate appraisal of the findings was possible only by considering the high dependency of the results on issues such as the national context under study, the family type examined, and the type of gamete used. These characteristics were recorded with the help of a data extraction sheet. Codes of each sub-category were correlated with characteristics of the studies as possible factors impacting on recipients’ motives and experiences and the results were reported.

## 3 Results

### 3.1 Identification and Selection of Studies

**FIGURE 1:**
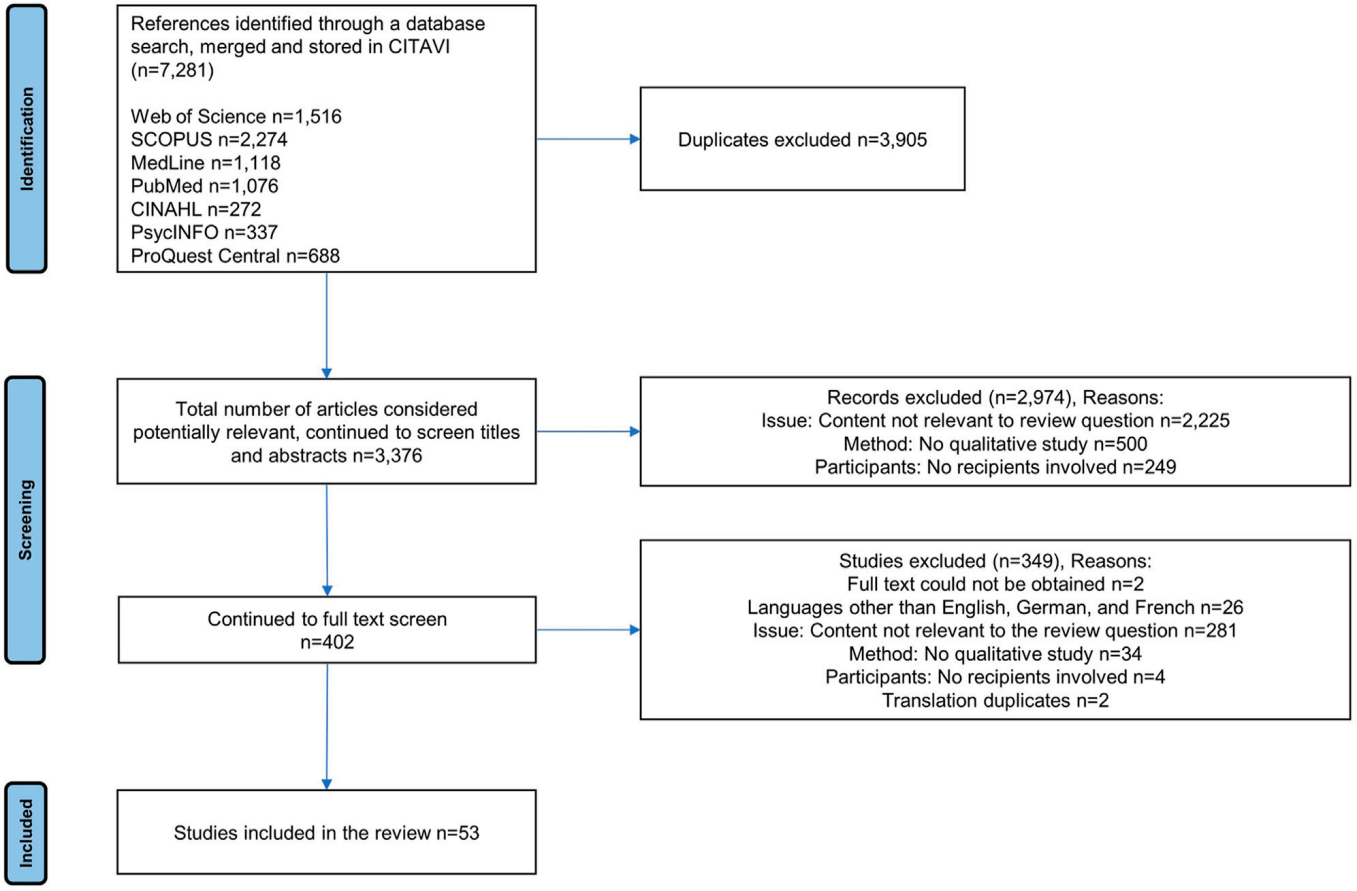
PRISMA flow chart of the literature search and screening process.

The search identified 7,281 references in all. After removing duplicates, 3,376 studies were screened for relevance in a two-stage procedure: first by title and abstract (2,974 studies excluded) and second by full text (349 studies excluded). Ancillary search strategies (manual search of major journals and forward/backward citation chaining of key references) were performed, but did not lead to the identification of additional studies to be included in the review. The remaining 53 studies were included in the review. In six studies, the same three datasets were analyzed by different sets of authors. In two studies, the same dataset was presented by the same author. All these studies were included in the review as the different forms of publication (article and doctoral dissertation) resulted in distinct differences in the extent of materials presented and levels of analysis. The 53 studies included in this review therefore represent 49 datasets. The literature search and study selection process is indicated using a PRISMA flow chart (Figure 1).

### 3.2 Characteristics of Selected Studies

The selected studies showed a diverse spectrum of characteristics (Supplementary File S2). The studies were published between 1988 and 2019, predominantly as academic articles in journals in fields like reproductive medicine, psychology, sexology, law, social sciences, queer studies, or bioethics (*n* = 45), and as PhD dissertations (*n* = 5) and book chapters (*n* = 3). Further, two studies were published in French, one in German, and 50 in English. The countries of study included the US (*n* = 15), the United Kingdom (*n* = 8), Belgium (*n* = 8), Canada (*n* = 6), France (*n* = 2), Germany (*n* = 2), and Israel, Sweden, and the Netherlands (*n* = 1 each). Some focused on multiple countries in the context of online surveys (*n* = 4), or on forms of CBRS (*n* = 4). One compared the donor choices of recipients in Sweden and Ireland (Ryan-Flood, 2005). The topic of motives and experiences of recipients choosing anonymous gamete donation was treated differently in the studies. Only a few focused exclusively on preferences for donor types (e.g., Brewaeys et al., 2005), whereas most treated the topic either as a part of their research question in a broader context such as the whole decision-making process on the route toward parenthood (e.g., Chabot, 1998), in conjunction with related topics such as disclosure decisions (e.g., Greenfeld and Klock, 2004), or as one of their findings—a theme emerging from the data (e.g., Kranz and Daniluk, 2006).

Most (*n* = 42) studies focused on sperm donation, and fewer on oocyte donation (*n* = 11). Two studies on sperm donation also included some cases of combinations of sperm and oocyte donations (Graham, 2014; Harrigan et al., 2017). Participants who had chosen anonymous and non-anonymous donations were included in the majority of studies (*n* = 33) while other participants were exclusively recipients of anonymous (*n* = 19) or open-identity (*n* = 1) donations. All studies were conducted in specific contexts determined by certain national legal frameworks or institutional regulations that restricted the actual reproductive options available to the participants and/or had a significant influence on the choice of donor. Changing legislation concerning donor anonymity in many countries made it imperative to clarify these circumstances diligently at the time and place of data collection for each study. These are reported in Supplementary File S2.

The studies were conducted before (*n* = 5), during (*n* = 3), and after treatment (*n* = 32), and some included participants at different stages of treatment (*n* = 13). In most of the studies conducted post-treatment, the donor-conceived children had not yet reached adolescence; the maximum age of the children reported was 29 years (Harrigan et al., 2017). The studies showed a wide range of sample sizes, from a one-participant case study (Marvel, 2013) to online surveys with the number of respondents exceeding 200 (Frith et al., 2012; Jadva et al., 2009). To counter difficulties in recruiting a sufficient number of participants for studies on such an intimate topic, which, in many cases, is related to issues of stigmatization, secrecy, and legal concerns, most studies relied on some form of convenience or self-selection sampling. The use of personal networks and support groups in combination with snowball sampling were identified as a frequent pattern of recruitment (e.g., Gartrell et al., 1996). By announcements on the Internet and social media, and through organizations like the Donor Sibling Registry, the researchers succeeded in recruiting large numbers of respondents, especially for online surveys (e.g., Frith et al., 2012; Blyth et al., 2013). Studies conducted in clinical settings were often linked to pre-treatment counseling and reported comparably high numbers of participants (e.g., Brewaeys et al., 2005; Baetens et al., 2000). For example, a study at the fertility clinic of a university hospital including 135 recipient couples used the interview data of pre-treatment psychological counseling as all patients had given their consent for the use of their clinical data for scientific purposes during admission (Laruelle et al., 2011). The participants in the included studies comprised heterosexual and lesbian recipients, as well as couples and single people (including single mothers by choice). Most studies limited their participants to certain family types or gender identities, whereas some included the attitudes of different groups of participants (e.g., Frith et al., 2012), or explicitly conducted a comparative inquiry between different family types (e.g., Brewaeys et al., 1993; Wendland et al., 1996; Jacob et al., 1999).

Most studies took a cross-sectional approach, except for seven, which were reported as being part of longitudinal investigations (Gartrell et al., 1996; Jacob et al., 1999; Brewaeys et al., 1995; Donovan and Wilson, 2008; Gartrell et al., 2015; Moget and Heenen-Wolff, 2015; Vanfraussen et al., 2001), and one which conducted multiple interviews over a one-year period to monitor changes in attitude and decision-making (Graham, 2014). Methods of data collection ranged from interviews (in some cases in the context of treatment-related counseling) and (online) surveys/questionnaires to observation, case studies, and fieldwork. Some studies sought to include the perspectives of non-biological parents in their investigation, for example, by separately interviewing both partners of a recipient couple, whereas other studies conducted conjoint interviews and focused on the couples’ joint decision-making processes. One exclusively explored the attitudes and preferences of non-biological parents (Frith et al., 2012). The quality criteria for the studies are reported in Table 1.

**TABLE 1:** Quality criteria.

Study: author(s), year	Was there a clear statement of the aims of the research?	Is the qualitative methodology appropriate?	Was it reported that the data were transcribed verbatim (i.e., were audiotapes, videotapes, or field notes used)?	If interviews were conducted, was it reported that the questions were predefined?	Was it reported that piloting interviews/focus groups or pretests were conducted?	Was there a description of how the research themes were identified?	Was it reported that the research findings were analyzed by more than one assessor?	Were the participant answers reviewed for clarification (i.e., member check)?	Were sequences from the original data presented (i.e., quotes)?	Did the study report ethical review and ethical waiver, respectively?
Almack, (2006)	+	+	−	−	−	−	−	−	+	−
Back and Snowden, (1988)	+	+	+	−	−	−	−	−	+	−
Baetens et al. (2000)	+	+	−	+	−	−	−	−	−	−
Bergmann, (2011)	+	+	−	−	−	−	−	−	+	−
Bertrand-Servais et al. (1993)	+	+	−	+	−	−	−	−	+	−
Blyth et al. (2013)	+	+	n/a	n/a	−	−	+	−	+	+
Brewaeys et al. (2005)	+	+	+	+	−	+	+	−	−	−
Brewaeys et al. (1995)	+	+	−	+	+	−	−	−	−	−
Brewaeys et al. (1993)	+	+	+	+	−	+	+	−	−	−
Burr, (2009)	+	+	+	+	−	+	−	−	+	+
Chabot and Ames, (2004)	+	+	+	+	−	+	−	+	+	−
Chabot, (1998)	+	+	+	+	+	+	−	+	+	−
Chamouard and Cohen de Lara, (2019)	+	+	−	+	−	+	−	−	+	−
de Melo-Martín et al. (2018)	+	+	+	+	−	+	+	−	+	+
Donovan and Wilson, (2008)	+	+	+	+	−	+	+	−	+	−
Frith et al. (2012)	+	+	n/a	n/a	−	−	+	−	+	+
Gartrell et al. (2015)	+	+	+	+	−	+	+	−	+	+
Gartrell et al. (1996)	+	+	+	+	+	+	+	−	+	−
Graham, (2014)	+	+	−	−	−	−	−	−	+	−
Greenfeld et al. (1998)	+	+	−	+	−	−	−	−	−	−
Greenfeld and Klock, (2004)	+	+	n/a	n/a	−	−	−	−	+	+
Gross and Richardot, (2019)	+	+	+	+	−	+	−	−	+	−
Haimes and Weiner, (2000)	+	+	+	+	−	−	−	−	+	−
Harrigan et al. (2017)	+	+	+	+	−	+	+	−	+	+
Haynes, (2003)	−	n/a	+	−	−	−	−	+	+	−
Herrmann-Green and Gehring, (2007)	+	+	n/a	n/a	−	+	−	−	+	−
Herrmann-Green and Herrmann-Green, (2008)	+	+	n/a	n/a	−	−	−	−	+	−
Hershberger, (2007)	+	+	+	+	−	+	−	+	+	+
Hershberger, (2005)	+	+	+	+	−	+	+	+	+	+
Hertz and Ferguson, (1997)	+	+	+	−	−	−	−	−	+	−
Jacob et al. (1999)	+	+	n/a	n/a	−	−	−	−	−	+
Jadva et al. (2009)	+	+	n/a	n/a	+	−	−	−	+	+
Kelly, (2012)	+	+	−	−	−	−	−	−	+	−
Kranz and Daniluk, (2006)	+	+	+	+	−	+	−	+	+	−
Kranz, (2005)	+	+	+	+	−	+	−	+	+	−
Landau and Weissenberg, (2010)	+	+	+	+	−	−	−	−	+	−
Laruelle et al. (2011)	+	+	−	+	−	−	+	−	−	−
Leblond de Brumath and Julien, (2012)	+	+	+	+	−	+	+	−	+	−
Leeb-Lundberg et al. (2006)	+	+	−	+	−	−	−	−	+	+
Marvel, (2013)	+	+	−	−	−	−	−	+	+	−
Moget and Heenen-Wolff, (2015)	+	+	−	−	−	+	−	−	+	−
Nordqvist, (2012)	+	+	+	+	−	+	−	−	+	+
Ryan-Flood, (2005)	+	+	−	−	−	−	−	−	+	−
Scheib et al. (2000)	+	+	−	+	−	−	−	−	−	+
Schwartz, (2010)	+	+	+	+	+	+	+	+	+	+
Smith, (2005)	+	+	+	+	+	+	+	+	+	−
Somers et al. (2017)	+	+	+	+	+	+	+	−	+	+
Stuart-Smith et al. (2012)	+	+	+	+	−	+	−	−	+	+
Touroni and Coyle, (2002)	+	+	+	+	−	+	+	−	+	−
Vanfraussen et al. (2001)	+	+	−	+	−	−	−	−	+	−
Vaughan, (2007)	+	+	−	−	−	−	−	−	+	−
Wendland et al. (1996)	+	+	n/a	n/a	−	−	−	−	−	−
Wyverkens et al. (2014)	+	+	+	+	−	+	+	−	+	+
total	52/53	52/53	28/53	34/53	7/53	26/53	16/53	10/53	44/53	18/53

### 3.3 Dimensions of Recipients’ Motives and Experiences in Relation to the Preference for Anonymous Gamete Donation

The data analysis identified 22 categories of recipients’ motives and experiences in relation to the preference for anonymous gamete donation. These categories cover various desired, anticipated, or experienced psycho-sociological benefits that the recipients associated with donor anonymity, with sub-categories describing the concrete means, facilitated by donor anonymity, through which the recipients experienced or intended to control, regulate, and or protect inter-stakeholder relations. The categories were further grouped into four main deductively introduced categories based on which of the stakeholders the recipients intended to keep ignorant of donor identity (or the child’s identity): the recipients themselves, the child, the donor, or the nuclear family’s social environment. A fifth main category covered pragmatic reasons underlying the recipients’ preference for donor anonymity. The main categories, categories and sub-categories are summarized in Table 2 and are explained in detail and illustrated by example quotes in the following sections.

**TABLE 2:** Dimensions of recipients’ motives underlying a preference for donor anonymity.

Main category	Category	Sub-category
Intention to keep oneself ignorant of donor identity	Create distance from the donor	Avoid considering the donor a person
		Minimize the donor’s contribution to the recipients’ parenthood
		Avoid psychological closeness with the donor
	Allow for imagining a benign, non-threatening donor figure	
	Avoid the emotional challenges of using a known donor	Alleviate feelings of indebtedness toward the donor
		Avoid the emotional impact of asking for help
		Avoid the discomfort that comes from using a known donor/family member as a donor
	Preclude the donor from interfering in establishing feelings of normality	Alleviate thoughts on infertility and feelings of inadequacy
		Alleviate feelings of one’s family deviating from the standard
		Avoid being reminded of the fact of donor treatment
	Prevent the donor from disturbing the recipient couple’s relationship	Prevent the donor from intruding into the recipient couple’s relationship
		Avoid fantasies of adultery between the donor and the biological parent
	Allow for full and truthful disclosure to the child	Ease disclosure of the mode of conception to the child
		Allow for full disclosure of available information to the child
		Avoid being blamed by the child for not disclosing donor information
	Enable the stabilization of parental feelings	Avoid disturbance of parental feelings toward the child
		Ensure the acceptance of the child by the non-biological parent
Intention to keep the child ignorant of donor identity	Create distance between the donor and the child	Preclude the child from being able to find or contact the donor
		Consider the child’s having information about the donor as not important
		Consider the genetic relationship between the child and the donor as not relevant
	Ensure the well-being of the child	Allow the child to create a positive image of the donor
		Provide the child with a clear and stable situation
		Save the child the anxiety over whether or not to contact the donor
	Protect the child from the harmful consequences of seeking to contact the donor	Protect the child from being disappointed by the donor
		Protect the child from being rejected by the donor
	Allow the consolidation of the recipients’ parentage	Prevent the child from picturing and personalizing the donor as a parent figure
		Avoid the child’s rejection of the non-biological parent
	Protect the donor	
Intention to keep the donor ignorant of the child’s identity	Preclude harmful consequences of the donor interfering in the child’s life	
	Preclude legal claims over the child by the donor	
	Prevent the interference of the donor in parenting issues	Prevent parental claims by the donor
		Ensure exclusive parental status
		Preclude challenges to the parental legitimacy of the non-biological parent by the donor
	Establish the feeling of being in control in family affairs	Avoid interference by the donor in family life
		Ensure family autonomy
		Establish clear family boundaries
		Realize own family ideals
	Ensure long-term stability of the family	
Intention to keep the family’s social environment ignorant of donor identity	Maintain secrecy and privacy	Maintain secrecy toward the social environment
		Ensure family privacy
	Avoid conflicts within the extended family (in case a known donor were to be used)	Avoid family conflict
		Avoid challenges to the legitimacy of the recipient couple’s relationship
		Avoid doubts regarding the parental status of the non-biological parent in the perception of the extended family
		Avoid conflicts in role-perception of extended family members in relation to the child
Pragmatic reasons	Lack of alternative options	Legal or regulatory constraints
		Unavailability of a suitable known donor
		Lack of awareness of other options
	Priority of treatment-related aspects over donor type	Higher medical success rate of treatment
		Priority of donor characteristics
		Priority of fast treatment
		Financial reasons
		Medical safety concerns
	Compliance with third-party preferences	

A considerable proportion of motives for choosing anonymous donation pertained to the recipients’ intention to remain themselves ignorant of the donor’s identity. As reported by the studies reviewed, by choosing not to know the donor’s identity, recipients expected to protect their psychological well-being and their relationship with their partners by controlling the distance to the donor. They also aimed to unburden their relationship with the child. These motives are summarized in the first main category.

Recipients perceived donor anonymity as a means to create and maintain a psychological distance from the donor. By remaining unaware of the identity of the donor, recipients could avoid acknowledging the donor as a person, classifying them as “not a real person” or a “non-person” (Burr, 2009:710) and were enabled to minimize the donor’s contribution to their family and parenthood: “We think that anonymity is to be taken into account in the denial of the genetic aspect of the oocyte. This genetic aspect is minimized and even sometimes ignored, not only by the woman but also by her partner. In this way the contribution of the husband’s spermatozoa is enhanced as is the pregnancy itself” (Bertrand-Servais et al., 1993:877). Thus, psychological closeness was avoided and the donor was kept at bay, avoiding jeopardizing the recipients’ perceptions of themselves and their families: “Distance was successfully created between recipients and donor as most women reported not giving the donor much thought and being content with not having met him” (Herrmann-Green and Gehring, 2007:386). Donor anonymity is further assessed as pivotal in creating a void that recipients can fill with images of the donor as a non-threatening figure, “that is more benign and has positive attributes” (Burr, 2009:715). The studies included also mentioned the recipients’ expectations around avoiding emotional challenges that would arise if they were to use a known donor. Donor anonymity helped alleviate “the debt of the recipient couple to the donor” (Baetens et al., 2000:482) and the donor’s contribution to the creation of the recipients’ new family and spared the recipients from “the emotional aspects of asking [potential known donors, T.B.] for help” (Laruelle et al., 2011:385). Recipients preferred anonymous donors in order to avoid awkwardness, “squeamishness and discomfort” (Schwartz, 2010:64), which come up in particular where a family member is to be the gamete donor.

Ignorance of the donor’s identity also helped recipients to establish a sense of normality. Recipients considered donor anonymity a benefit as it allowed them to repress or alleviate thoughts about their infertility, as “a known donor could be an unwanted reminder” of the recipients’ infertility (Stuart-Smith et al., 2012:2,069), and feelings that their family deviates from the standard, as some recipients “saw access to the donor’s identifiable information as a reminder of their families’ differences, and therefore experienced anonymity as protective” (de Melo-Martín et al., 2018:239). Donor anonymity also allowed them to avoid being reminded of the fact of donor treatment (de Melo-Martín et al., 2018:239). Maintaining distance from the donor through anonymity was considered useful in preventing especially a known donor from disturbing and (psychologically) intruding into the recipient couple’s relationship (Stuart-Smith et al., 2012:2,070) and in warding off troubling fantasies of adultery between the donor and the biological parent: “Anonymity also protects against the fantasy of the husband’s adultery; a fear commonly expressed is that the wife might have the impression that her husband had a child by her sister. By choosing anonymity, couples are removing the aspect of sex from the donor” (Bertrand-Servais et al., 1993:877).

The data showed that anonymity allowed recipients to make a full and truthful disclosure to the child by revealing all information available on the mode of conception and the donor. Donor anonymity made the disclosure of the fact of donor conception to the child easier for the recipients (Landau and Weissenberg, 2010:944). It relieved them of possible conflicts regarding whether to share potentially disruptive identifying information about the donor with the child and allowed for full disclosure of all information available to them: “Often their [lesbian couples as recipients, T.B.] rationale for opting for [anonymous, T.B.] DI [donor insemination, T.B.] was reiterated as a reason for the story they could tell: the fact that they did not know the identity of the donor meant they would be freed up to tell the children exactly what they knew in the security and safety of knowing that there was no more to be known” (Donovan and Wilson, 2008:657). Thus recipients “saw anonymity as a way to avoid being blamed by their children” for not disclosing donor identity (de Melo-Martín et al., 2018:245). Ignorance of donor identity also contributed toward stabilizing the recipients’ parentage by avoiding disturbances to their parental feelings toward the child: “One couple described an unanticipated benefit to using an anonymous donor; Jackee and Kelly noted that because they did not know the donor or even see a picture of him before insemination, they were more able to see the twin girls they conceived as solely their own” (Schwartz, 2010:68, 69). Donor anonymity was further considered beneficial in ensuring the non-biological parent’s acceptance of the child (Wendland et al., 1996:767).

The second main category comprised the motive to keep the child ignorant of donor identity. Motives and experiences in this category were concerned with protecting the well-being of the child by controlling the donor-child relationship. They also included motives concerned with the child-recipient relationship and protection of the donor.

The studies showed that recipients considered donor anonymity as a means to create distance between the donor and their child. Donor anonymity is seen as a guarantee that precludes the child from contacting the donor: “[A]nonymous donation made it impossible for the child to be able to find or contact the donor, which was a comforting thought for some parents” (Somers et al., 2017:15). The recipients justified donor anonymity on the grounds that “knowing the donor’s identity would not improve the child’s well-being and could even be harmful” (Brewaeys et al., 2005:822), and that it was not necessary for the child to possess identifiable information on the donor. At a more fundamental level, they questioned the relevance of the genetic relationship between the child and the donor, as this was “insignificant in comparison to parentage based on everyday caring for the child and concern for its needs” (Landau and Weissenberg, 2010:946). Recipients believed that donor anonymity would enable a child to cope with the existence of a donor and the fact of being donor-conceived and therefore ensure the child’s well-being. The reasons provided for this included the assumptions that donor anonymity allows a child to “create a positive image of the donor” (Somers et al., 2017:15), provides a child with a clear and stable family situation (Donovan and Wilson, 2008:655), and saves the child “from the anxiety over whether or not to contact their donor” (de Melo-Martín et al., 2018:243). Anonymity was also described as a means of protecting the child from potentially harmful consequences of seeking contact with the donor, which may result in disappointment: “Reasons against choosing the donor I.D. [identity release, T.B.] program for some of the participants included a fear that the image of a father figure a child could carry with them could be shattered 18 years from now, and that they did not want to put their child through that” (Chabot, 1998:88, 89). “[T]o protect the child from a sense of rejection, i.e., should the donor not be traceable or not want contact, etc.” was another rationale for recipients to choose anonymous donations (Hermann-Green and Gehring, 2007:388).

Recipients also indicated that the child-recipient relationship benefits from donor anonymity, as it allows recipients to consolidate their parentage. Keeping a child ignorant of the donor’s identity is believed to prevent the child from picturing and personalizing the donor as a parent figure: “Discovering information about the donor entails the risk that it will become a big issue for the children, as she [the recipient, T.B.] uses the term ‘father’. She claims that the construct of the donor will change from ‘cell’ to ‘person’ to ‘father’ as soon as the children know more about him. Therefore, she clearly states her preference for the anonymous system” (Wyverkens et al., 2014:1,252). Donor anonymity was further considered beneficial in preventing the child from rejecting the non-biological parent: “[P]articipants saw anonymity as a way to lessen anxieties about their child’s possible rejection. The fact that the donor’s identity is unknown provides reassurance to mothers that their children will not replace them as mothers” (de Melo-Martín et al., 2018:240). Two studies reported the “protection of the donor” (Baetens et al., 2000:479), “releasing him from any obligation” (Somers et al., 2017:15), as a motive for choosing anonymous donation.

The third main category comprised motives and experiences centered on the ignorance of the donor, as the third stakeholder (the other two being the recipients and the child). In contrast to the intentionality of ignorance described in the previous category, here recipients’ anticipated or actual benefits revolve around the donor’s ignorance of the child’s identity. The main motives associated with this form of ignorance were expectations of being able to control the influence of the donor on the child and the child’s well-being, on the recipients’ parenting, and on family affairs, thus ensuring long-term stability of family. A large part of the motives and experiences summarized in this main category are related to the recipients’ decision to avoid a known donor, as this donor type would entail immediate issues of donor involvement and legal dimensions, which were often perceived by the recipients as undesirable, threatening, or as a potential source of conflict. However, accounts of recipients to whom identity-release donors were available as an alternative to anonymous donation (e.g., Scheib et al., 2000) also fell into this main category, as they reported on recipients’ anticipation of future issues that could arise upon the donor’s getting into contact with the recipients’ families if this type of donor were used.

Precluding donor interference in child-related issues was frequently identified in the studies included. Donor anonymity was considered by recipients to protect the child from harmful consequences of the donor interfering in the child’s life, such as the disruption of the stability of a child’s family environment by introducing a “potentially harmful multiple-parent situation” (Baetens et al., 2000:482) and to preclude legal claims on the child as recipients were concerned “that a non-anonymous donor could seek legal rights” (Frith et al., 2012:714). Further, the donor’s ignorance of the child’s identity can prevent the donor’s interference with parenting, for example in the form of parental claims by the donor, as recipients “preferred anonymity because of a concern that the donor ‘would want to participate in the parenting’ with a fear that that participation would complicate their relation to the child” (Greenfeld and Klock, 2004:1,567). Donor anonymity was considered to ensure the recipients’ exclusive parental status (Chabot, 1998:86), and to circumvent challenges to the parental legitimacy of the non-biological parent by the donor: “Lesbians who have chosen [anonymous, T.B.] DI to create their families use the enforced secrecy of ‘unknown’ donors to protect the family unit from intrusion by outsiders. … This is particularly the case regarding perceived interference of biological fathers into their children’s lives, thereby limiting or disavowing their own parental rights, and especially those of the social mother” (Smith, 2005:63). The donor’s ignorance allowed recipients to establish a feeling of being in control of their family affairs as such ignorance helped avoid “interference by a third party” in their family life (Vanfraussen et al., 2001:2,023), ensured family autonomy (Harrigan et al., 2017:284), “protect[ed] family boundaries” (Hermann-Green and Gehring, 2007:388), and enabled the recipients to realize their family ideals, such as “a vision of joint parenting and family without the contingencies of donor involvement” (Donovan and Wilson, 2008:655). Keeping the donor ignorant of the child’s identity or avoiding “long-term insecurity in a known donor situation” (Stuart-Smith et al., 2012:2,070) was also seen as a guarantee to ensure lasting stability of the family.

The fourth main category focused on keeping the family’s social environment ignorant of the donor’s identity. The motives underlying this category pertained on the one hand to maintaining secrecy and privacy against the social environment of the family in general, and on the other to avoiding conflict with the extended family in particular, which may have arisen if a known person, especially a family member were to have been used as donor. The studies included showed that recipients preferred anonymous donation to keep the circumstances of their child’s conception a secret from those in their social environment (Laruelle et al., 2011:385). Donor anonymity was closely linked to the recipients’ desire for “the privacy of the family” (Vanfraussen et al., 2001:2,023). Some studies pointed out that ignorance of donor identity served as a means to avoid conflicts with their extended family. This category was exclusively comprised of accounts of the preferences of lesbian couples, whose choice for anonymous sperm donation was primarily motivated by their intention to circumvent conflicts expected to arise if a known donor, especially a family member, were to be used, for example in the case of the brother of the co-mother acting as sperm donor: “The plan to use brothers as donors was rejected by couples for various reasons including … the recognition that family relationships could become strained as a result” (Schwartz, 2010:64). Some studies reported that recipients preferred anonymous donors as they expected this to reduce the likelihood of family conflict or challenges to the legitimacy of the parent couple’s relationship by members of the extended family (Vaughan, 2007:147). Studies also reported that recipients considered donor anonymity an advantage in that it helped to avoid both doubts about the parental status of the non-biological parent among the extended family and conflicts in role-perception among the extended family in relation to the child (Kranz, 2005:61).

The fifth main category comprising pragmatic reasons focused on motives and experiences pertaining to donor anonymity that were not directly grounded in expectations arising out of anonymity, that is, expected benefits from keeping certain stakeholders in ignorance. Rather, it focused on secondary aspects accompanying anonymous donations. This category comprised various factors. The studies included revealed that the lack of alternatives to anonymous donation left some recipients with no choice other than to opt for an anonymous donation. One reason for this could be legal or regulatory constraints such as “the sperm bank they went to did not offer non-anonymous donors or it was contrary to regulations in their country” (Frith et al., 2012:713), or the unavailability of known donors: “But not all women actually choose: some would prefer a known donor, but unable to find a known donor are forced to settle for a unknown donor” (Hertz and Ferguson, 1997:203). The lack of awareness of other options was another reason identified by the studies included (Jadva et al., 2009:179). These recipients chose anonymous donation because they did not have or were not aware of other alternatives.

Some recipients reported that they preferred an anonymous donation not based on an explicit consideration of benefits of anonymity per se, but rather because of medical, financial, and other aspects linked to an anonymous donation. Narratives mentioned in the literature showed that some recipients chose anonymous donations because of the accompanying advantages, such as a higher medical success rate of anonymous treatment (Laruelle et al., 2011:385). Other recipients prioritized specific donor characteristics, that is they “were not concerned about the identity-release status of the donor, but chose their donor on the basis of the donor’s personal and medical characteristics” (Frith et al., 2012:713) or preferred faster treatment that the use of anonymous donors allowed (Scheib et al., 2000:54). Financial reasons underlying the choice for an anonymous donation were also reported: “The move to anonymous donation was not prompted by a shift in the importance placed on the donor and information about him but due to a perceived inability to continue treatment using identity-release sperm donation. Financial considerations were key for three of the participants: they felt forced to seek substantially cheaper treatment abroad” (Graham, 2014:220). Medical safety concerns, in particular medical screening of sperm provided by sperm banks, were mentioned as one reason lesbian couples and single mothers by choice opted against a known donor and chose anonymous donation: “The major positive aspects of this choice [for anonymous donation, T.B.] included safety (sperm that were tested for HIV, STDs)” (Herrmann-Green and Gehring, 2007:386). Some studies mentioned that recipients chose anonymous donations in compliance with a third party’s preferences, such as their partner’s strongly preferring an anonymous donation, the request of the donor recruited to a known-anonymous program by the recipient, or compliance with assumed expectations among lesbian peers to ensure their acceptance (Haimes and Weiner, 2000:481).

### 3.4 Factors Impacting Recipients’ Motives and Experiences Related to Anonymous Donation

Correlating the various motives and experiences in relation to donor anonymity with characteristics of the studies as possible factors impacting the relevance of particular motives and experiences for certain recipients in specific situations did not lead to conclusive results in form of distinct patterns being identified. The analysis rather showed that the majority of motives and experiences were relevant for example to both recipients of sperm and oocyte donation, and to both lesbian and heterosexual recipients. However, the following observations could be made.

Some motives and experiences identified in this review pertained less to the choice *for* an anonymous donor than to the choice *against* other donor types, in particular against a known donor, such as the motives and experiences of the main category “intention to keep the donor ignorant of the child’s identity” or the “intention to keep oneself ignorant of donor identity” in order to “avoid the emotional challenges of using a known donor.” This highlighted the importance of the alternatives to anonymous donation available to recipients in a specific context for their choice of donor type (may this be the result of practical, legal, or institutional circumstances). Further, the fact that only heterosexual couples were identified as preferring to keep themselves ignorant of donor identity in order to “preclude the donor from interfering in establishing feelings of normality” and solely lesbian couples chose anonymous donors to “avoid conflicts within the extended family” arguably points to social expectations, family norms, and notions of infertility forming the background against which the recipients of these specific family types lay out their respective strategies of family making. Additionally, the fact that the “intention to keep the child ignorant of donor identity” in order to “protect the child from the harmful consequences of seeking to contact the donor” was almost exclusively identified by studies on sperm donation might suggest that recipients tend to have differing images of sperm and oocyte donors concerning their openness and willingness to attend to the needs of their genetic children in case these choose to contact them. Finally, the impact of social and legal circumstances on the recipients’ motives for anonymous donation is reported for example by a longitudinal study by Gartrell et al. (2015), suggesting that the “intention to keep the donor ignorant of the child’s identity” in order to “preclude legal claims over the child by the donor” stems from a situation of legal (un)certainty and a lack of legal recognition of lesbian families, and can become obsolete in places and times when such legal protection is installed.

## 4 Discussion

The results showed a broad of spectrum of motives and experiences on the part of recipients in relation to donor anonymity. They also pointed out some limitations of the existing research and helped identify topics and approaches for future research. These areas will be discussed in this section in detail. The implications and limitations of this review and its methodology will also be addressed critically.

### 4.1 Current State of Research and Perspectives for Future Inquiry

#### 4.1.1 Change in Recipients’ Attitudes Over Time

The integrated overview of the studies included in this review strongly suggests that recipients’ motives and preferences pertaining to donor anonymity are not necessarily fixed and unchangeable, but are subject to reconsideration and revision over time. Some studies framed the recipients’ pre-treatment considerations of donor types as an ongoing and multifaceted process, in which initially preferred and imagined routes to parenthood can be abandoned in favor of alternatives, such as by reconsidering and gradually replacing an original preference for a known donor with the eventual decision to use an anonymous one (Donovan and Wilson, 2008). Pre-treatment preferences were reported to evolve and transform in the face of post-treatment experiences. While some studies reported recipients satisfied with anonymous donation (Gartrell et al., 2015), a significant number of recipients who had conceived through an anonymous donation, indicated that they regretted the decision in retrospect (e.g., Brewaeys et al., 1995; Blyth et al., 2013; Gartrell et al., 2015).

Only some studies explicitly expounded on the change in the recipients’ attitudes over time. This review was able to identify a number of studies incorporating (or being part of) a longitudinal approach. However, most studies restricted their analysis to the preferences and experiences of recipients at a specific point in time. Understanding the recipients’ needs and preferences concerning the desire to know or not know—not only the donor’s identity, but also donor information in general (Stuart-Smith et al., 2012)—as constantly evolving through various stages of treatment and family life (such as pre-treatment stage, pregnancy, postnatal stage, subsequent stages of parenting, tackling the question of disclosure, adolescence of the child, etc.) is an important aspect that future studies must consider, as this area needs more detailed research. Those studies identified by this review as constituting longitudinal projects focused exclusively on lesbian families (Gartrell et al., 1996; Jacob et al., 1999; Brewaeys et al., 1995; Donovan and Wilson, 2008; Gartrell et al., 2015; Moget and Heenen-Wolff, 2015; Vanfraussen et al., 2001). This highlights the dearth of such approaches for other forms of families, such as those of heterosexual couples or single mothers by choice. Longitudinal studies can help overcome some limitations of the studies reviewed that emerged from the retrospective accounts of the recipients on the rationale for their initial decision around donor anonymity, such as the “birth reconstructive bias,” that is a modification of the parents’ initial accounts and perspectives through their subsequent experience of childbirth (Donovan and Wilson, 2008; Leblond de Brumath and Julien, 2012). In particular, future researchers could focus on the issue of which motives for an initial preference for donor anonymity became less important to the recipients over time, which motives and concerns remained important or even gained momentum, and which initially anticipated benefits of ignorance concerning donor identity could be replaced by alternative compensating measures. Findings on these areas could profoundly inform the practice of recipients’ counseling accompanying ART treatment through gamete donation, and highlight the need to provide counseling services to recipients both in the pre-treatment stage and in later stages of their family-building endeavors (Wyverkens et al., 2014).

#### 4.1.2 Perspective of the Non-biological Parent

As demonstrated by this review, the intention to protect the parental status of the non-biological parent is a recurring motive underlying the preference for anonymous donation. However, motives and experiences of non-biological parents in relation to sperm donor anonymity remain under-researched. Most studies on DI settings either did not explicitly collect data from non-biological parents (e.g., Stuart-Smith et al., 2012; de Melo-Martín et al., 2018; Hershberger, 2007), or did not systematically analyze and report on the differences in the attitudes between biological, and non-biological parents concerning sperm donor anonymity (e.g., Laruelle et al., 2011; Ryan-Flood, 2005; Harrigan et al., 2017). Studies that involved the recipients’ partners in many cases employed joint interviews as a means of data collection, which, especially in cases involving retrospective perspectives on the decision-making process, made it difficult to screen out the perspectives of non-biological parents because of a possible “rehearsed” and “co-constructed” nature of the couples’ accounts (Donovan and Wilson, 2008). Although a joint interview approach could be justified by the assumption that “this format would convey a recognition of the collaborative nature of the decision-making” (Touroni and Coyle, 2002:196), studies that relied on methods of separate data collection tended to identify the asymmetries in the biological and non-biological parents’ attitudes toward donor anonymity in DI scenarios more clearly (e.g., Brewaeys et al., 1995; Leblond de Brumath and Julien, 2012; Leeb-Lundberg et al., 2006). Focusing exclusively on the non-biological parents’ motives and experiences in relation to donor anonymity (Frith et al., 2012) is highly instructive for future research that can contribute further toward the understanding of the commonalities, differences, and interpersonal dynamics of biological and non-biological parents’ views on donor anonymity.

#### 4.1.3 Data Comprehensiveness and Analytical Depth

As mentioned above, only some of the studies focused specifically on the questions pertaining to preferences regarding the donor type as formulated in the review question (Supplementary File S2, column “focus of study”). Therefore, especially in light of the detailed map of possible motives presented by this review (Table 2), details and nuances of the recipients’ motives and experiences with respect to donor anonymity were sometimes vague or ambiguous. This ambiguity was observed on two levels. First, the presented data itself, such as interview excerpts, exhibited ambiguity. The rationale underlying the preference for anonymous donation exemplified by the recipients’ statements such as, “that it would have been easier for the father” (Leeb-Lundberg et al., 2006:80) left ample room for interpretation in the context of this review. Second, the analysis and presentation of some studies’ findings in relation to the recipients’ motives and experiences concerning donor anonymity remained vague for the purpose of this review. For example, summaries of motives for anonymous donations under labels such as “[m]inimize the link between the donor and the child” (Baetens et al., 2000:479) could pertain to one or more of any of the broad range of motives in main categories two and three in this review. Therefore, inquiries employing detailed approaches to data collection by considering the entire spectrum of motives for anonymous donation as presented by this review, along with a corresponding thorough and differentiated analysis can significantly enhance the understanding of the recipients’ motives and experiences. This is one of the key contributions of the categorization developed by this review (Table 2), as it not only raises awareness of the dimensions of potential motives, but also the related delimitations, nuances, directions, and intentions.

### 4.2 Implications and Limitations of This Review

#### 4.2.1 Reframing the Discourse on Donor Anonymity From the Perspective of Ignorance

The approach chosen for this review led to the introduction of four main categories of motives and experiences pertaining to the intention to keep the recipients themselves, the child, the donor, or the family’s social environment in ignorance of the donor’s identity (or, in the case of the donor, the child’s identity). A closer examination of the contents of each of these main categories shows that the intention to keep a certain stakeholder in ignorance is not limited to controlling just this specific stakeholder. Most forms of ignorance seem to have been employed with the aim (or at least the potential) of simultaneously influencing multiple stakeholders and relationships among them. For example, categories summarized under the main category “intention to keep the child ignorant of donor identity” relate to issues of protecting the child and controlling the relationship between the donor and the child by creating a distance between them, and to matters of donor protection and benefiting the relationship between the recipients and the child.

This finding points to alternatives to common framings of the discussion on donor anonymity in terms of competing rights (the child’s right to know their genetic origins vs. the parents’ right to privacy and autonomy) (Frith, 2001; Herbrand and Hudson, 2015). From the perspective of ignorance, as proposed by this review, the interests of recipients and the child seem far less diametrically opposed, and the choices and preferences of recipients appear to have also anticipated and incorporated (their understanding of) the future needs and welfare of the child. The preference for anonymous donation by recipients can thus be interpreted in a more positive light, that is, as the employment of a different strategy as a choice for a known donation while also considering the interests and the welfare of the child (Greenfeld and Klock, 2004:1,570).

Further, more than the terms “anonymity” and “secrecy” convey, the term “ignorance” underscores the fact that a significant proportion of the recipients’ preference for donor anonymity is related to the intention to keep the recipients themselves unaware of the donor’s identity, as shown in the first main category. This also puts into perspective frameworks that aim to reduce donor anonymity to the recipients’ intention to keep the child ignorant and to place the child’s infringed right to know in opposition to the recipients’ interests.

#### 4.2.2 Context Dependency of Motives and Experiences Pertaining to Donor Anonymity

Although this review—consistent with its aim to carve out the entire unabridged spectrum of possible motives and experiences involved—was able to collate data from the current literature, the findings presented are limited by the fact that motives and experiences are strongly influenced by the particular circumstances of the participants involved in each individual study. The decision to include studies on both sperm and oocyte donation and on recipients of various sexual orientations and forms of partnership in this review contributed to a thorough understanding of the full spectrum of motives and experiences concerning donor anonymity in general. Although relevant features of the studies reviewed, such as the time of assessment, the types of donors and gametes, and the socio-legal background, were thoroughly reported in this review (Supplementary File S2) and were correlated with the established framework (Section 3.4), it was precisely this wide range of differing situations, and a multitude of other relevant interwoven factors that diminished the possibilities of a comprehensive comparative analysis and a far-reaching synthesis of the findings, which is reported as a hindering factor of data integration in reviews conducted on related topics (e.g., Indekeu et al., 2013). Future research could therefore attempt to re-apply the dimensions of the recipients’ possible motives and experiences pointed out by this review to specific contexts of gamete donation and use it as a framework to identify commonalities and differences in the patterns of motives and preferences of recipients in more specific contexts.

Although some of the studies reported on participants’ sociodemographic characteristics such as religion or ethnicity and inquired into the influence of these factors on recipients’ choices of anonymous donation (e.g., Brewaeys et al., 2005; Laruelle et al., 2011), future research could further explore more deeply such societal and cultural factors presumably underlying recipients’ strategies of family-making and parenting in the context of a preference for anonymous gamete donation. One example would be the impact of religion and sectarian differences on choices regarding anonymous donors. This seems all the more important as the studies included in this review almost exclusively focused on Western European and North American countries. Broadening the circle of countries to test the cross-cultural applicability of this reviews’ approach to frame motives and experiences in relation to donor anonymity as a eufunctional form of ignorance could contribute to further refine or modify this framework. Such research could contribute to our understanding of how for example culture-specific notions of infertility, family norms, or concepts of kinship impact on the recipients’ anticipated or experienced benefits of ignorance.

Another direction for future research comprises investigations of contexts that allow recipients to exert their choices concerning donor anonymity in situations that are comparatively free of legal constraints. Such a free and unrestricted choice between anonymous and non-anonymous donors in double-track systems or in non-clinical settings such as Internet-facilitated sperm donations (Jadva et al., 2018) seems promising in the pursuit of deepening the understanding of the role of donor anonymity in the recipients’ reproductive decisions.

#### 4.2.3 Ignorance of Donor Identity and Related Information

This review was limited to a specific kind of information concerning the donor that the recipients preferred to keep themselves or others ignorant of, namely the donor’s identity. However, donor identity is not the only information that recipients have to deal with. Even where anonymous donations are made, recipients relying on sperm banks may have access not only to basic biometric information on the donor, but also to an extensive range of additional non-identifying information such as their “background, physical characteristics, education, profession, personality, health and family history, photos, handwritten greeting, voice sample, staff impression, and EQ tests” (Cryos International, 2021). In such cases, recipients must identify the extent to which they want to or do not want to know, or let their offspring or the family’s social environment know such information. As some of the studies included suggest (Stuart-Smith et al., 2012; Wyverkens et al., 2014), some motives identified by this review, especially those grouped in the first main category “intention to keep oneself ignorant of donor identity” also apply to such non-identifying information. For example, the recipients’ desire to “minimize the donor’s contribution to the recipients’ parenthood” can serve as an underlying motivation not only for the choice of donor anonymity, but also for an attempt by the recipients to minimize their knowledge of the donor’s non-identifying information (Stuart-Smith et al., 2012). Therefore, future research should strive to investigate recipients’ attitudes toward donor anonymity in the broader context of their preferences and strategies in managing information pertaining to their ART treatment through gamete donation in general, including disclosure to the child. This can deepen our understanding of the decisions that recipients of gamete donation must make.

## 5 Conclusion

This systematic review provided an overview of the spectrum of possible motives and experiences of recipients in relation to a preference for anonymous gamete donation by examining the findings of qualitative studies. To interpret these motives and experiences as a form of ignorance directed toward particular stakeholders involved allows the reframing of the discourse on donor anonymity; this is a fruitful approach that can be refined further and applied in future research. Potential directions for future research on motives for donor anonymity as identified by this review include the need for more thorough inquiries into the change in recipients’ preferences over time, such as by making comparisons of pre- and post-treatment attitudes toward donor anonymity in the form of longitudinal studies, and focusing on the perspective of non-biological parents.

## Data Availability

The original contributions presented in the study are included in the article/Supplementary Material, further inquiries can be directed to the corresponding author.
