# Supplementary material for: A Systematic Review of Qualitative Studies Investigating Motives and Experiences of Recipients of Anonymous Gamete Donation
Source: Front Sociol. 2022 Feb 16;7:746847. doi: 10.3389/fsoc.2022.746847 (PMC8889113; doi:10.3389/fsoc.2022.746847)
Supplement: Supplementary file 2 [file Table1.docx]

| basic search syntax | ((*anonym* OR *know* OR *identi* OR secre*) AND (gamete OR sperm OR egg OR oocyte OR ovum OR insemin* OR reproduc*) AND (donor* OR donat*) AND (prefer* OR view* OR attitude* OR choice* OR experience* OR motivation* OR opinion* OR decision* OR perception* OR expectation* OR psychosocial OR survey OR questionnaire OR qualitative OR interview*)) | |
| --- | --- | --- |
| database | search syntax | number of references identified  (total: 7,281) |
| Web of Science (Core Collection 1900-present) | TS=((*anonym* OR *know* OR *identi* OR secre*) AND (gamete OR sperm OR egg OR oocyte OR ovum OR insemin* OR reproduc*) AND (donor* OR donat*) AND (prefer* OR view* OR attitude* OR choice* OR experience* OR motivation* OR opinion* OR decision* OR perception* OR expectation* OR psychosocial OR survey OR questionnaire OR qualitative OR interview*)) | 1,516 |
| SCOPUS | TITLE-ABS-KEY((*anonym* OR *know* OR *identi* OR secre*) AND (gamete OR sperm OR egg OR oocyte OR ovum OR insemin* OR reproduc*) AND (donor* OR donat* ) AND (prefer* OR view* OR attitude* OR choice* OR experience* OR motivation* OR opinion* OR decision* OR perception* OR expectation* OR psychosocial OR survey OR questionnaire OR qualitative OR interview*)) AND (EXCLUDE(EXACTKEYWORD, "Nonhuman")) | 2,274 |
| MedLine (Web of Science) | TS=((*anonym* OR *know* OR *identi* OR secre*) AND (gamete OR sperm OR egg OR oocyte OR ovum OR insemin* OR reproduc*) AND (donor* OR donat*) AND (prefer* OR view* OR attitude* OR choice* OR experience* OR motivation* OR opinion* OR decision* OR perception* OR expectation* OR psychosocial OR survey OR questionnaire OR qualitative OR interview*)) [Refined by: MeSH HEADINGS: ( HUMANS )] | 1,118 |
| PubMed | ((*anonym*[Title/Abstract] OR *know*[Title/Abstract] OR *identi*[Title/Abstract] OR secre*) AND (gamete[Title/Abstract] OR sperm[Title/Abstract] OR egg[Title/Abstract] OR oocyte[Title/Abstract] OR ovum[Title/Abstract] OR insemin*[Title/Abstract] OR reproduc*) AND (donor*[Title/Abstract] OR donat*) AND (prefer*[Title/Abstract] OR view*[Title/Abstract] OR attitude*[Title/Abstract] OR choice*[Title/Abstract] OR experience*[Title/Abstract] OR motivation*[Title/Abstract] OR opinion*[Title/Abstract] OR decision*[Title/Abstract] OR perception*[Title/Abstract] OR expectation*[Title/Abstract] OR psychosocial[Title/Abstract] OR survey[Title/Abstract] OR questionnaire[Title/Abstract] OR qualitative[Title/Abstract] OR interview*)) AND Humans[Mesh] | 1,076 |
| CINAHL | TI(((*anonym* OR *know* OR *identi* OR secre*) AND (gamete OR sperm OR egg OR oocyte OR ovum OR insemin* OR reproduc*) AND (donor* OR donat*) AND (prefer* OR view* OR attitude* OR choice* OR experience* OR motivation* OR opinion* OR decision* OR perception* OR expectation* OR psychosocial OR survey OR questionnaire OR qualitative OR interview*))) OR AB(((*anonym* OR *know* OR *identi* OR secre*) AND (gamete OR sperm OR egg OR oocyte OR ovum OR insemin* OR reproduc*) AND (donor* OR donat*) AND (prefer* OR view* OR attitude* OR choice* OR experience* OR motivation* OR opinion* OR decision* OR perception* OR expectation* OR psychosocial OR survey OR questionnaire OR qualitative OR interview*))) | 272 |
| PsycINFO (OVID 1806 to December Week 4 2019) | ((anonym* or know* or identi* or secre*) and (gamete or sperm or egg or oocyte or ovum or insemin* or reproduc*) and (donor* or donat*) and (prefer* or view* or attitude* or choice* or experience* or motivation* or opinion* or decision* or perception* or expectation* or psychosocial or survey or questionnaire or qualitative or interview*)).mp. [mp=title, abstract, heading word, table of contents, key concepts, original title, tests & measures, mesh] | 337 |
| ProQuest Central | noft(((anonym* OR know* OR identi* OR secre*) AND (gamete OR sperm OR egg OR oocyte OR ovum OR insemin* OR reproduc*) AND (donor* OR donat*) AND (prefer* OR view* OR attitude* OR choice* OR experience* OR motivation* OR opinion* OR decision* OR perception* OR expectation* OR psychosocial OR survey OR questionnaire OR qualitative OR interview*))) [Additional limits: Document type: Article, Bibliography, Book, Book Chapter, Case Study, Conference Paper, Conference Proceeding, Evidence Based Healthcare, Literature Review, Statistics/Data Report, Working Paper/Pre-Print] | 688 |
| search conducted: 03/01/2020 | | |
